# Vertically distinct patterns of soil carbon and nitrogen stock depletion associated with Moso bamboo encroachment into subtropical forests

**DOI:** 10.3389/fpls.2026.1954514

**Published:** 2026-09-30

**Authors:** Jianhong Xu, Lei Hua, Wenjie Long, Juan Liu, Dongnan Hu, Songze Wan

**Affiliations:** 1 College of Forestry, Jiangxi Agricultural University, Jiangxi Provincial Key Laboratory of Improved Variety Breeding and Efficient Utilization of Native Tree Species, Nanchang, China; 2 Jiangxi Provincial Key Laboratory of Subtropical Forest Resource Cultivation, Nanchang, China

**Keywords:** functional genes, *Phyllostachys edulis*, plant encroachment, soil degradation, soil priming effect

## Abstract

Moso bamboo (*Phyllostachys edulis*) expansion is strongly associated with soil degradation in subtropical forests, but the underlying abiotic and biotic factors regulating soil organic carbon (SOC) and total nitrogen (TN) stocks across different stands remain unclear. Here, we investigated depth-dependent (0–40 cm) changes in SOC and TN stocks, alongside the abundances of autotrophic (*cbbL*) and denitrifying (*nosZ*) functional genes, associated with bamboo encroachment into broad-leaved (BLF) and coniferous forests (CF). While bamboo invasion consistently depleted SOC and TN pools across the entire 0–40 cm soil profile in both stands, the most severe relative losses exhibited distinct depth-dependent patterns linked to divergent ecological pathways. In the BLF, the prominent stock depletions in the topsoil (0–10 cm) were jointly associated with a hypothesized litter-induced soil priming effect and the physical degradation of the surface microenvironment. Conversely, in the CF, the most severe stock reductions occurred in the sub-soil layer (20–40 cm), where shifts in soil bulk density and moisture likely weakened the physical protection of legacy organic matter and potentially exacerbated resource limitations for deep-soil microbes. Concurrently, the decline in *cbbL* and *nosZ* gene abundances served as sensitive potential microbial indicators reflecting these severe environmental stresses. These findings underscore the necessity of depth-explicit and stand-tailored strategies for mitigating soil degradation, and suggest that incorporating belowground functional genes could improve future plant invasion risk assessments in subtropical ecosystems.

## 1 Introduction

As a dominant species widely distributed across the Chinese subtropics, Moso bamboo (*Phyllostachys edulis*) possesses high productivity and a short growth cycle, providing substantial economic values such as edible shoots and timber resources (Qiu et al., 2021; Wu et al., 2024). However, the progressive encroachment of this clonal species into neighboring stands has caused serious ecological problems in local forest communities (Song et al., 2016; Fang et al., 2022a). In recent decades, this expansion has become a critical environmental issue in East Asia, with the total bamboo forest area in China now exceeding 7.5 × 10^6^ ha (Fang et al., 2022a). Although economically beneficial, Moso bamboo expansion often leads to native ecosystem degradation, including soil erosion (Fukushima et al., 2015), inhibited seedling growth (Larpkern et al., 2011), and biodiversity loss (Tomimatsu et al., 2011; Lima et al., 2012). Therefore, Moso bamboo expansion is increasingly regarded as a biotic factor associated with land degradation that decreases soil quality and impairs forest C and N sequestration (Lin et al., 2025; Meng et al., 2025).

Recent research suggests that the initial stand type largely determines the ecological consequences of bamboo encroachment. For example, encroachment upon broad-leaved stands suppresses soil N mineralization by altering litter quality (Lin et al., 2025), whereas invading coniferous forests can increase labile N pools and accelerate organic matter degradation by soil microbes (Wu et al., 2017; Fang et al., 2022b). Consequently, the responses of soil organic carbon (SOC) and total nitrogen (TN) stocks to bamboo encroachment remain highly debated, with previous studies reporting conflicting results ranging from stock accumulation to significant depletions (Fukushima et al., 2015; Li et al., 2017; Fang et al., 2022b). These inconsistent responses may be related to initial soil properties and litter characteristics. In broad-leaved forests with lower initial C/N ratios, bamboo litter input can induce a strong soil priming effect and accelerate organic matter decomposition (Luo et al., 2024). Conversely, in coniferous forests with higher initial C/N ratios, extensive bamboo root inputs may intensify belowground resource competition and alter soil C and N dynamics (Yan et al., 2025). Currently, how these divergent pathways distinctly impact specific soil depths, and the underlying abiotic and biotic factors regulating these depth-dependent processes, remain poorly understood (Liu et al., 2019).

Soil microorganisms are essential drivers of soil C and N transformations (Li J. L. et al., 2025). Specifically, functional microbial groups carrying the *cbbL* gene (involved in autotrophic C fixation) and the *nosZ* gene (involved in denitrification) serve as sensitive markers reflecting soil nutrient availability (Wang et al., 2023, Wang et al., 2024). Importantly, while macroscopic net SOC and TN stocks are primarily governed by the long-term balance between net physical inputs and heterotrophic decomposition, the abundances of these specific functional genes rapidly respond to local environmental stresses. For instance, the *cbbL* and *nosZ* communities are highly sensitive to shifts in soil pH, bulk density, and substrate availability induced by invasive species (Zhang et al., 2024; Meng et al., 2026). Moso bamboo expansion can reshape soil microbial community structure and alter functional gene abundances across distinct forest ecosystems (Lin et al., 2025; Wu et al., 2026). However, how these microbial indicators parallel shifts in basic soil properties to reflect the degradation of SOC and TN stocks remains unclear.

In this study, we selected broad-leaved (BLF) and coniferous forests (CF) undergoing Moso bamboo expansion to investigate depth-dependent changes in organic C and total N storage, alongside autotrophic (*cbbL*) and denitrifying (*nosZ*) functional gene abundances, throughout a 40 cm soil profile. Based on the disparities in initial stand properties and bamboo expansion strategies, we hypothesized that while bamboo invasion would broadly decrease SOC and TN pools across the 0–40 cm profile associated with aggressive system-wide resource uptake, the most severe depletions would exhibit distinct depth dependencies. Specifically, we hypothesized that (1) within broad-leaved stands, the stock reduction would be primarily concentrated in the topsoil, as the input of bamboo litter into an initially low C/N environment likely triggers a strong surface priming effect that accelerates legacy organic matter decomposition (Qin et al., 2017; Luo et al., 2024); and (2) within coniferous stands, the depletion would be more pronounced in the sub-soil, because the recalcitrant nature of coniferous litter limits surface priming, whereas the aggressive expansion of dense bamboo rhizome networks into deeper horizons intensifies belowground physical disturbance and root competition (Xiao et al., 2021; Lin et al., 2025).

## 2 Materials and methods

### 2.1 Study site and experimental design

Field sampling and observations were performed at the Dajing Forest Farm (26°34′7″N, 114°8′16″E), which lies within the Jiangxi Jinggangshan National Nature Reserve, China. The elevation of the study site averages approximately 975 m. The local climate is typically subtropical and monsoonal, bringing wet, hot summers along with humid, mild winters. The yearly mean temperature (MAT) fluctuations span from 15 to 17 °C, while the average annual rainfall (MAP) reaches nearly 1860 mm. Regarding the edaphic features, mountain yellow-lateritic substrates constitute the primary soil type across this area, exhibiting a high percentage of stony debris and gravel pieces.

In April 2022, four fixed quadrats (20 m × 20 m) were established in broad-leaved forests (BLF) and their adjacent bamboo-broadleaf mixed forests (BBLF), as well as in coniferous forests (CF) and their adjacent bamboo-coniferous mixed forests (BCF). To avoid edge effects, the distance between any two adjacent quadrats exceeded 20 m. These plots were established within a single continuous stand on the same mountain to minimize large-scale spatial heterogeneity. The progressive encroachment of Moso bamboo into these native stands has occurred over approximately 10 years (Lin et al., 2025). Prior to moso bamboo encroachment, the BLF and CF were well-preserved mature stands, with their dominant tree species, *Machilus thunbergii* and *Cryptomeria japonica*, accounting for approximately 85% and 82% of the total stand density, respectively (Lin et al., 2025). Following encroachment, the proportion of moso bamboo (*Phyllostachys edulis*) individuals reached approximately 76.2% in the BBLF and 69.7% in the BCF stands.

### 2.2 Soil sampling and analysis

Field sampling took place in July 2024. After clearing the surface litter, we randomly extracted nine soil cores (2.5 cm in diameter) from each plot. To reduce within-plot spatial heterogeneity, these nine cores were fully homogenized into a single composite sample for each of the three distinct soil layers (0–10 cm, 10–20 cm, and 20–40 cm). Consequently, the plot served as the independent statistical unit in our analysis (n=4 per forest type and depth). Furthermore, undisturbed soil samples were taken at each corresponding depth with 100-cm³ stainless steel cylinders to measure the soil bulk density (BD).

Upon arrival at the laboratory, we manually cleared the samples of visible plant roots, stones, and soil fauna. After passing through a 2-mm sieve, each composite sample was divided into three fractions for different downstream analyses. A fresh fraction was kept at 4 °C for DOC evaluation, while a second fraction was rapidly frozen at -80 °C for microbial functional gene (*cbbL* and *nosZ*) extraction. The third fraction was air-dried, crushed, and sieved through a 0.15-mm mesh to determine pH, SOC, and TN. For DOC determination, samples were mixed with deionized water (ratio of 1 to 20, w/v), shaken, filtered, and analyzed on a multi N/C 2000 analyzer (Analytik Jena, Germany). We measured soil pH using a standard pH meter in a soil-water mixture (ratio of 1 to 2.5, w/v). Additionally, the SOC and TN contents were assessed via the classic potassium dichromate oxidation and Kjeldahl acid digestion methods, respectively (Lin et al., 2025).

### 2.3 Genomic material isolation and real-time quantitative PCR

We extracted soil DNA from 0.5 g fresh samples using a commercial kit (Fujike Biological Technology Co., Ltd., Chengdu, China) following standard protocols. We verified DNA integrity through 1% agarose gel electrophoresis and measured its concentration with a D30 nucleic acid-protein detector. The extracted DNA was kept at -20 °C for subsequent experiments.

To determine the copy numbers of *cbbL* and *nosZ*, qPCR was performed according to Li X. A. et al. (2025). The template DNA was diluted to a 1 to 40 ratio, and each sample was tested with four replicates. We amplified the *cbbL* gene using the forward primer 5′-AAGGAYGACGAGAACATC-3′ and reverse primer 3′-TGCAGSATCATGTCRTT-5′. The *nosZ* gene was amplified with the forward primer 5′-AGAACGACCAGCTGATCGACA-3′ and reverse primer 3′-TCCATGGTGACGCCGTGGTTG-5′.

The total reaction volume was 20 μL, consisting of 10 μL TB GREEN Premix Ex Taq II, 0.8 μL of each primer, 0.4 μL ROX Reference Dye II, 1 μL DNA template, and 7 μL nuclease-free water. The PCR conditions for *cbbL* included an initial 30 s step at 95 °C and 40 amplification cycles (95 °C for 10 s, 53 °C for 30 s, and 72 °C for 30 s). The *nosZ* amplification involved 30 s at 95 °C initially, followed by 40 cycles comprising 95 °C for 15 s, 53 °C for 15 s, and 72 °C for 60 s. We constructed standard curves from 10-fold serial dilutions of recombinant plasmids (10³ to 10^8^ copies) to calculate the absolute gene abundances.

### 2.4 Calculations and statistical analyses

To evaluate SOC and TN storage, we adopted the standard formulation proposed by Guo and Gifford (2002), with the resulting *Stock* values expressed in Mg ha^-1^.


$$Stock\;=\frac{C\;\times \;BD\;\times \;D}{10}$$

In this formula, *C* signifies the specific C or N concentration (g kg^-1^). *BD* denotes the soil dry bulk density (g cm^-3^), and *D* indicates the thickness of the soil layer (cm).

Furthermore, to evaluate the magnitude of soil organic pool depletion following bamboo encroachment, the relative loss (%) of SOC and TN stocks at each specific depth was calculated using the following equation: *Relative loss* (%) = (*Stock_native_*-*Stock_mixed_*)/*Stock_native_* × 100, where *Stock_native_* represents the SOC or TN stock in the undisturbed native forests (BLF or CF), and *Stock_mixed_* represents the corresponding stock in the bamboo-encroached mixed forests (BBLF or BCF).

Additionally, to distinguish the underlying physical and chemical drivers of the observed stock depletions, we mathematically decomposed the total SOC and TN stock losses (Δ*Stock*) into the relative contributions of element concentration changes (Δ*C*) and soil bulk density changes (Δ*BD*). Based on the basic stock equation, an exact decomposition without residual terms was formulated using the mean values of the variables:


$$\Delta Stock\;=\frac{\Delta C\times BD_{\text{mean}}\times D}{10}+\frac{\Delta BD\times C_{\text{mean}}\times D}{10}$$

In this formula, *C*_mean_ and *BD*_mean_ signify the average values of concentration and bulk density between the native and mixed stands, respectively. Δ*C* denotes the absolute stock change driven solely by concentration, and Δ. *BD* indicates the stock change driven by bulk density. The relative percentage contribution of each factor was calculated as the ratio of its isolated effect to the total stock loss.

Statistical analyses were conducted using SPSS 21.0 (SPSS Inc., Chicago, IL, USA), and data were presented as means ± standard errors (SEs). Linear mixed-effects models (LMMs) were used to analyze the effects of forest type, soil depth, and their interaction on soil physicochemical properties, functional gene abundances, and carbon and nitrogen pools. In the models, forest type, soil depth, and their interaction were set as fixed effects. Meanwhile, the sampling plot was included as a random effect to account for the spatial dependence of different depths within the same plot. *Post-hoc* pairwise comparisons were conducted when significant effects were observed. Differences were considered statistically significant at *p<* 0.05. Furthermore, we employed Amos 28.0 (IBM, USA) to perform structural equation modeling (SEM). Instead of testing depth-specific mechanisms, the SEM was utilized to evaluate the overarching interconnected pathways among soil physicochemical properties, functional gene abundances, and carbon/nitrogen concentrations across the entire 0–40 cm profile. To avoid model overfitting given the sample size, non-significant paths were sequentially removed during model fitting to obtain a parsimonious and reliable final model. All figures were developed using Origin 2021.

## 3 Results

### 3.1 Responses of soil properties and functional genes in broad-leaved forests

Moso bamboo expansion into native broad-leaved forests (BLF) altered the soil physicochemical environment. Across the 0–40 cm profile, SWC and BD were significantly lower in the BBLF than in the undisturbed BLF, while soil pH significantly increased in the 0–20 cm layer (**Figures 1a, c, e**). At the same time, the concentrations of DOC, SOC, and TN decreased at all sampled depths (**Figures 2a, c, e**). Similarly, a downward trend was recorded in the abundance of soil *cbbL* and *nosZ* markers (**Figures 3a, c**).

**Figure 1:**
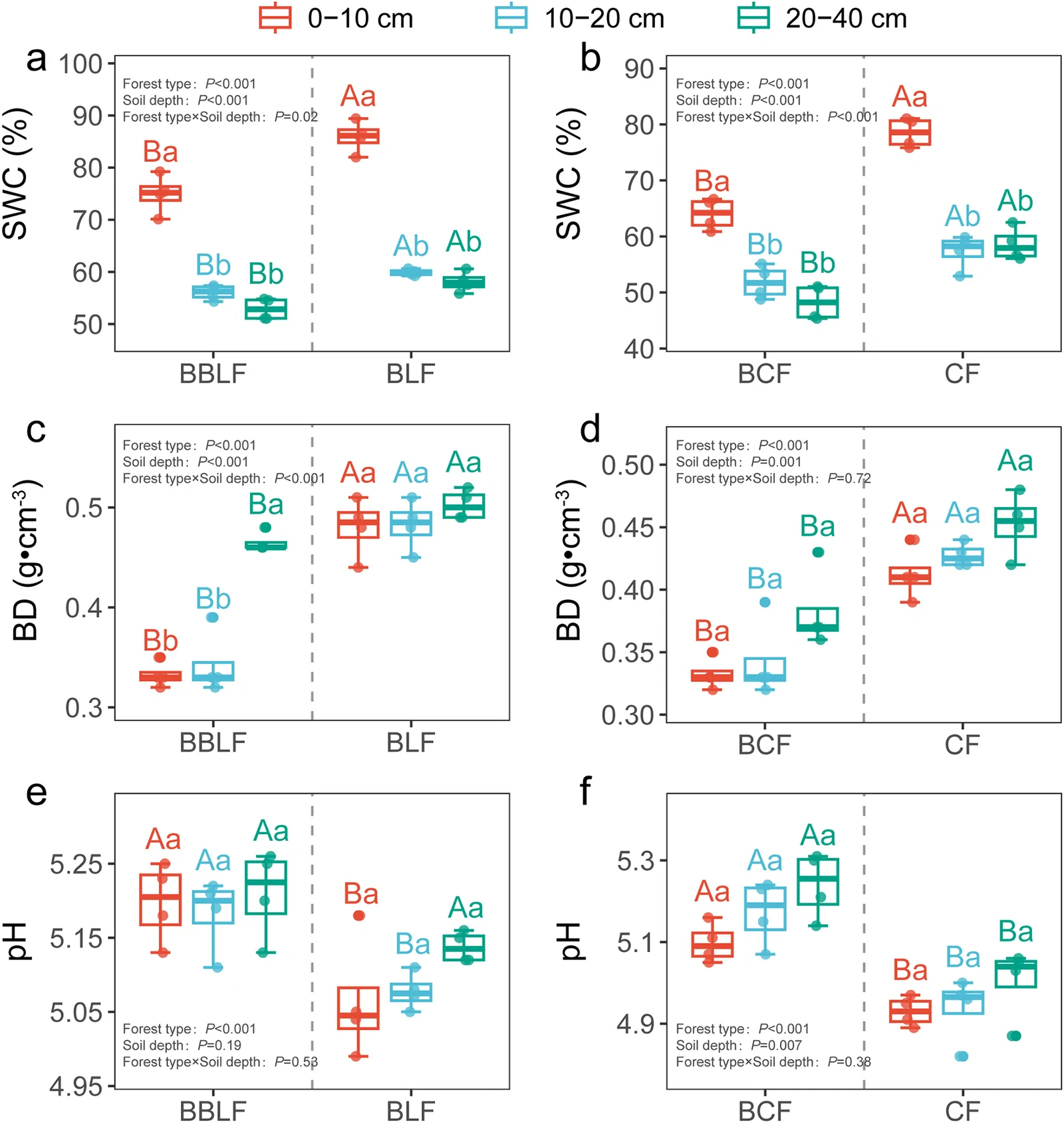
Variations of soil water content (SWC), bulk density (BD), and pH across soil depths under different forest types. **(a, c, e)** Bamboo-broad-leaved mixed (BBLF) vs. Broad-leaved forests (BLF); **(b, d, f)** Bamboo-coniferous mixed (BCF) vs. Coniferous forests (CF). Different uppercase letters indicate significant differences among forest types within the same soil layers (*p* < 0.05), while different lowercase letters denote significant differences among soil layers within the same forest type (*p* < 0.05). Boxes represent the 25th and 75th percentiles with median lines shown (n = 4 plots per forest type and depth), and dots indicate outliers. Linear mixed-effects models were used for statistical analyses.

**Figure 2:**
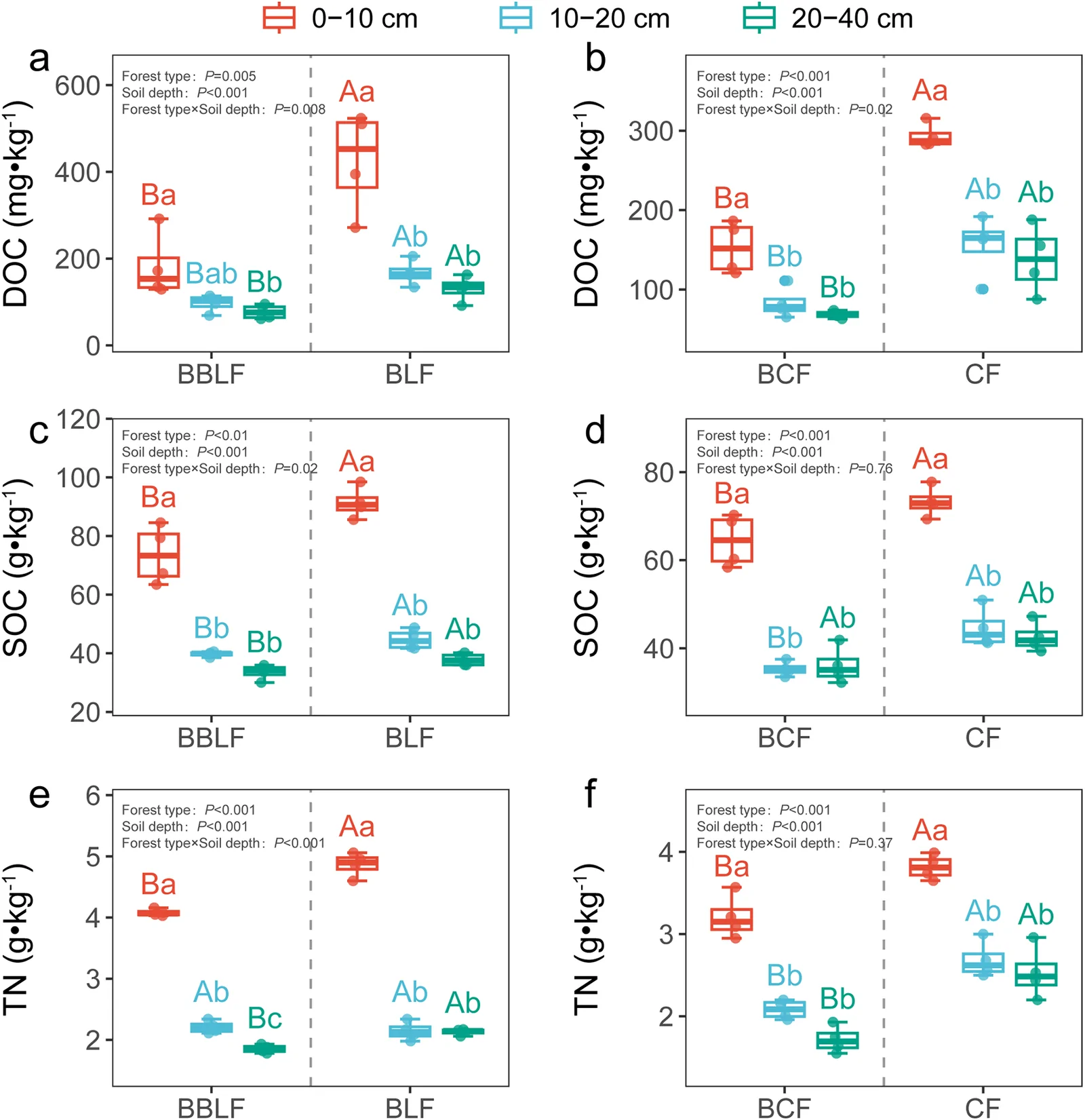
Variations of soil dissolved organic carbon (DOC), soil organic carbon (SOC), and total nitrogen (TN) contents across soil depths under different forest types. **(a, b)** DOC, **(c, d)** SOC, and **(e, f)** TN. See **Figure 1** for explanations of boxplots and statistical letters.

**Figure 3:**
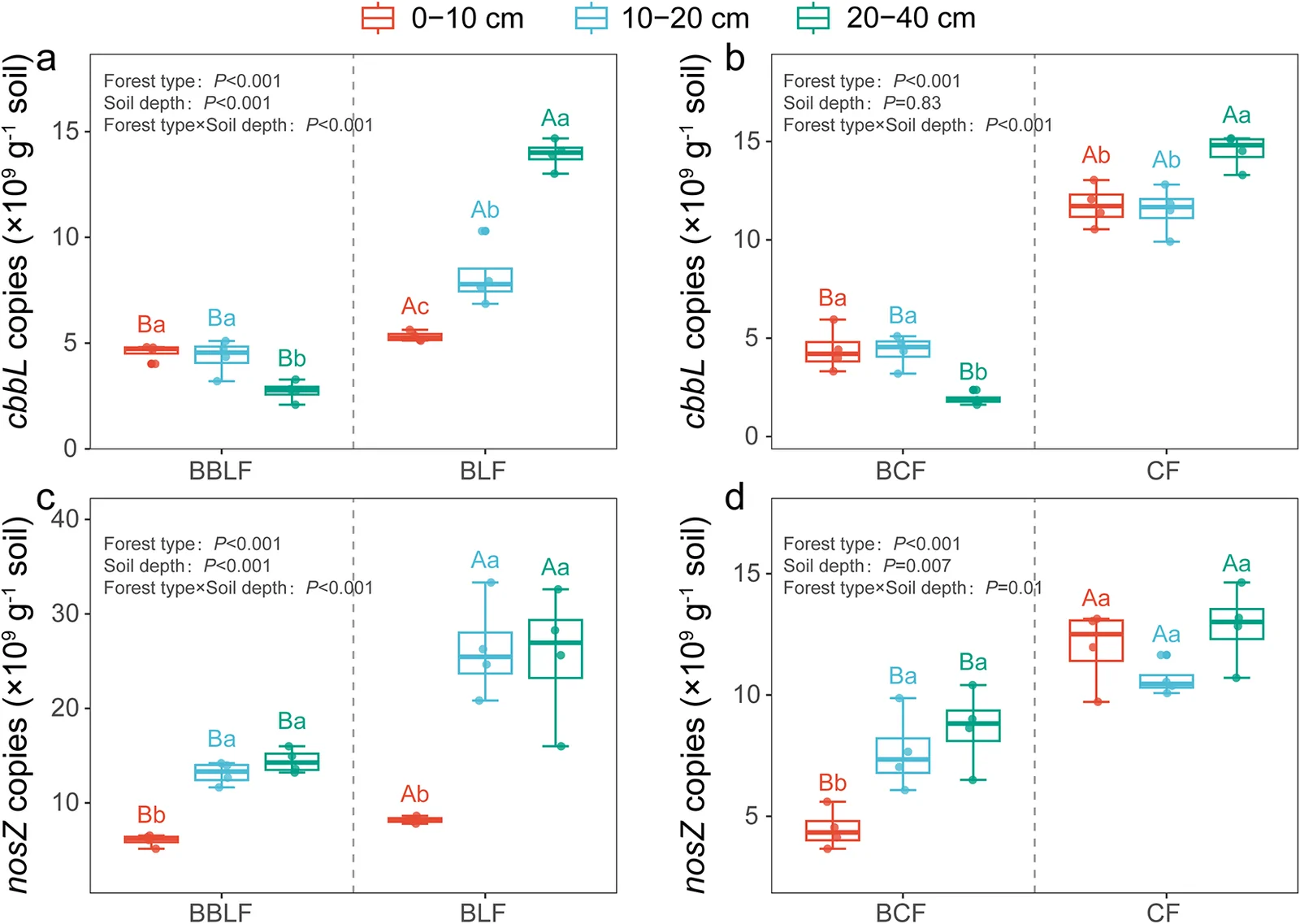
Variations of the copy numbers of bacterial genes *cbbL* and *nosZ* across soil depths under different forest types. **(a, b)** *cbbL* and **(c, d)** *nosZ*. See **Figure 1** for explanations of boxplots and statistical letters.

The depletion of SOC and TN pools extended across the entire 40 cm sampling depth. Notably, the highest relative losses of both SOC and TN stocks were concentrated within the 0–10 cm upper horizon, which were significantly greater than the proportional losses observed in the deeper soil layers (**Figures 4a, c**; **Table 1**). Quantitative decomposition further revealed that the decline in BD accounted for over 60% of these severe surface stock losses for both SOC and TN (**Supplementary Table S1**). According to the overarching SEM across the 0–40 cm profile, the variance in SOC concentrations was primarily driven by DOC (**Figures 5a, b**). Soil pH, SWC and BD exerted significant indirect effects on SOC concentrations, while *cbbL* gene abundance did not show a significant direct path. For the TN concentrations, the variance was significantly associated with soil BD (**Figures 5c, d**). The *nosZ* gene abundance, SWC, and soil pH did not show significant direct paths to TN concentrations.

**Figure 4:**
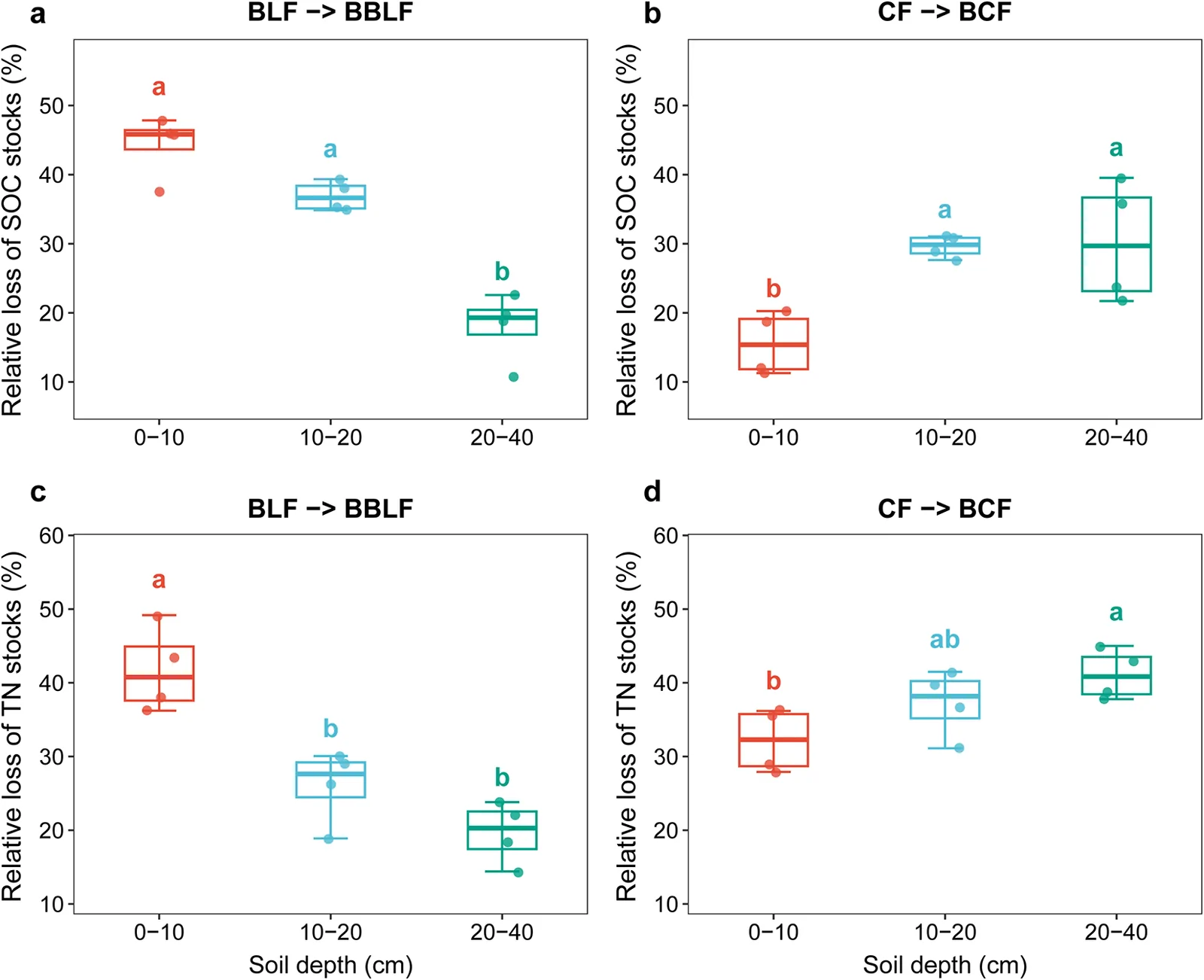
Relative loss (%) of SOC and TN stocks under different forest types as affected by soil depths. The relative loss was calculated as the percentage decrease in stocks from native forests (BLF or CF) to their corresponding bamboo-encroached mixed forests (BBLF or BCF). **(a, b)** SOC stocks and **(c, d)** TN stocks. See **Figure 1** for explanations of boxplots and statistical letters.

**Table 1:** Effects of Moso bamboo expansion on SOC and TN stocks across different depths.

Soil variable	Forest type	0–10 cm	10–20 cm	20–40 cm	Cumulative
SOC stocks (Mg·ha^-1^)	BBLF	24.36 ± 1.45B	13.60 ± 0.65B	31.27 ± 1.22B	69.23 ± 2.98B
	BLF	43.68 ± 1.02A	21.53 ± 0.82A	38.23 ± 1.53A	103.44 ± 2.1A
TN stocks (Mg·ha^-1^)	BBLF	1.35 ± 0.03B	0.76 ± 0.05B	1.72 ± 0.04B	3.83 ± 0.10B
	BLF	2.33 ± 0.08A	1.04 ± 0.04A	2.15 ± 0.03A	5.52 ± 0.11A
SOC stocks (Mg·ha^-1^)	BCF	25.40 ± 0.85B	13.48 ± 0.91B	26.45 ± 0.77B	65.33 ± 1.20B
	CF	30.14 ± 0.67A	19.12 ± 1.10A	38.44 ± 2.29A	87.70 ± 2.83A
TN stocks (Mg·ha^-1^)	BCF	1.06 ± 0.06B	0.72 ± 0.05B	1.31 ± 0.04B	3.09 ± 0.08B
	CF	1.57 ± 0.05A	1.15 ± 0.06A	2.29 ± 0.19A	5.01 ± 0.27A

Data are presented as means ± SE (n = 4). Different uppercase letters within the same column indicate significant differences between forest types at the same soil depth (*p<* 0.05). BLF, evergreen broad-leaved forest; BBLF, bamboo-broad-leaved mixed forest; CF, coniferous forest; BCF, bamboo-coniferous mixed forest.

**Figure 5:**
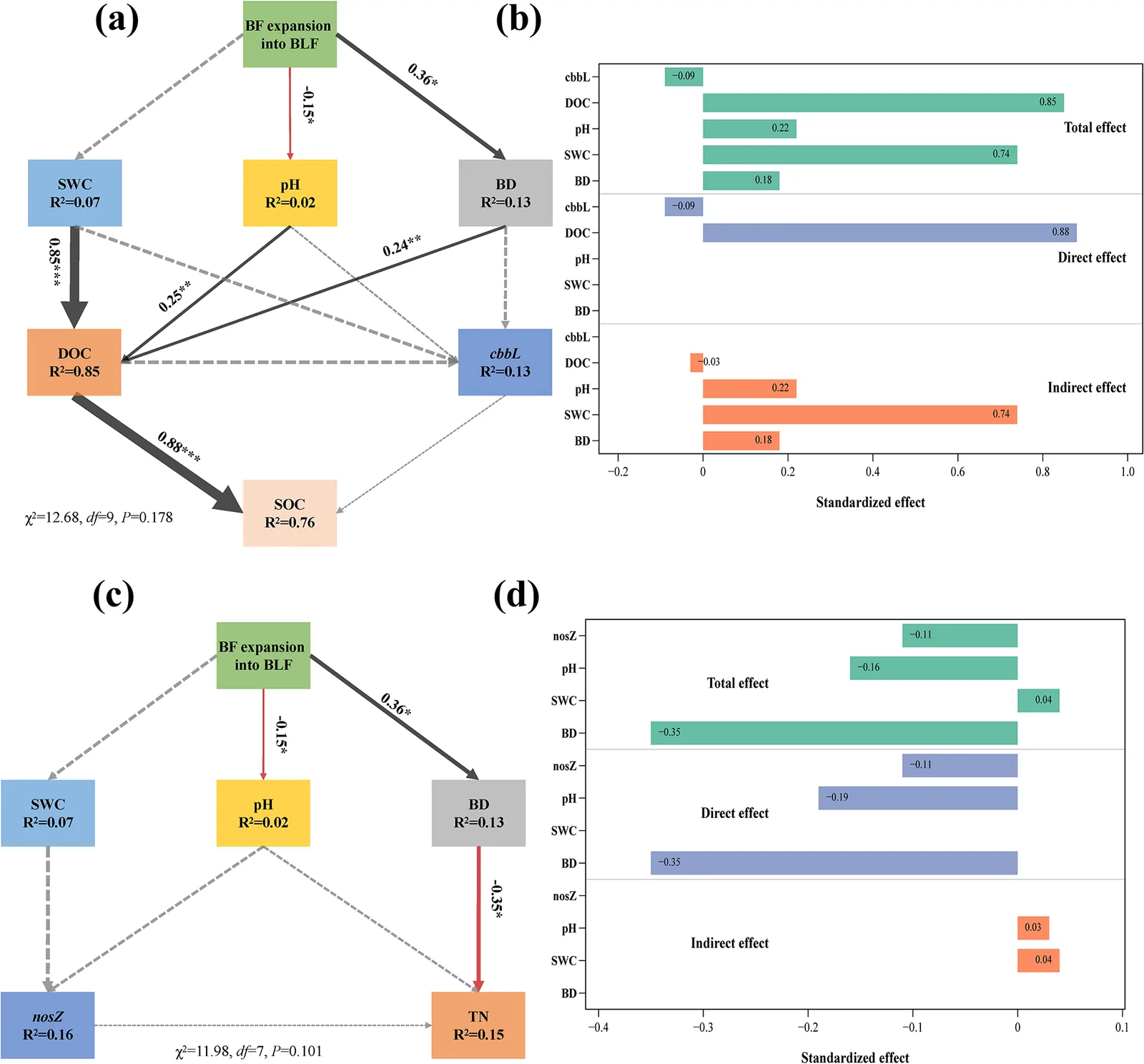
Structural equation modeling (SEM) illustrating the impacts of soil abiotic and biotic properties on SOC **(a)** and TN **(c)** concentrations during BF expansion into BLF. Statistical significance was tested using 24 independent soil samples. Solid black and red arrows indicate significant positive and negative effects (*p* < 0.05), respectively, and dashed arrows represent nonsignificant relationships. Values adjacent to arrows are standardized path coefficients, with arrow width proportional to coefficient magnitude. R^2^ donates the proportion of variance explained by the model. Significant levels for predictors: *p < 0.05, **p < 0.01, ***p < 0.001. Standardized total effects of individual drivers on SOC **(b)** and TN **(d)** concentrations derived from the SEM above.

### 3.2 Responses of soil properties and functional genes in coniferous forests

In coniferous forests (CF), Moso bamboo encroachment significantly reduced SWC, BD, and the contents of DOC, SOC, and TN across the 0–40 cm profile, while consistently elevating soil pH at all depths (**Figures 1b, d, f**, **2b, d, f**). These abiotic shifts were accompanied by a significant reduction in the abundances of both *cbbL* and *nosZ* genes (**Figures 3b, d**).

Regarding soil organic pools, both SOC and TN stocks decreased irrespective of soil depth. However, in stark contrast to the broad-leaved stands, the relative losses in the coniferous forests were most prominent in the deeper soil horizons. The 20–40 cm subsoil exhibited significantly higher percentage declines in both SOC and TN stocks compared to the 0–10 cm surface layer (**Figures 4b, d**). According to the quantitative decomposition, while the deep-soil SOC stock loss was jointly driven by concentration and bulk density shifts, the severe sub-soil TN depletion was predominantly driven by the decline in nitrogen concentration (**Supplementary Table S1**). Based on the SEM results for the entire soil profile, variations in SOC concentrations were closely related to *cbbL* gene abundance, with soil SWC and BD exerting indirect effects (**Figure 6**). Meanwhile, the decline in TN concentrations corresponded to the reduced *nosZ* gene abundance, which was indirectly influenced by soil SWC (**Figure 6**).

**Figure 6:**
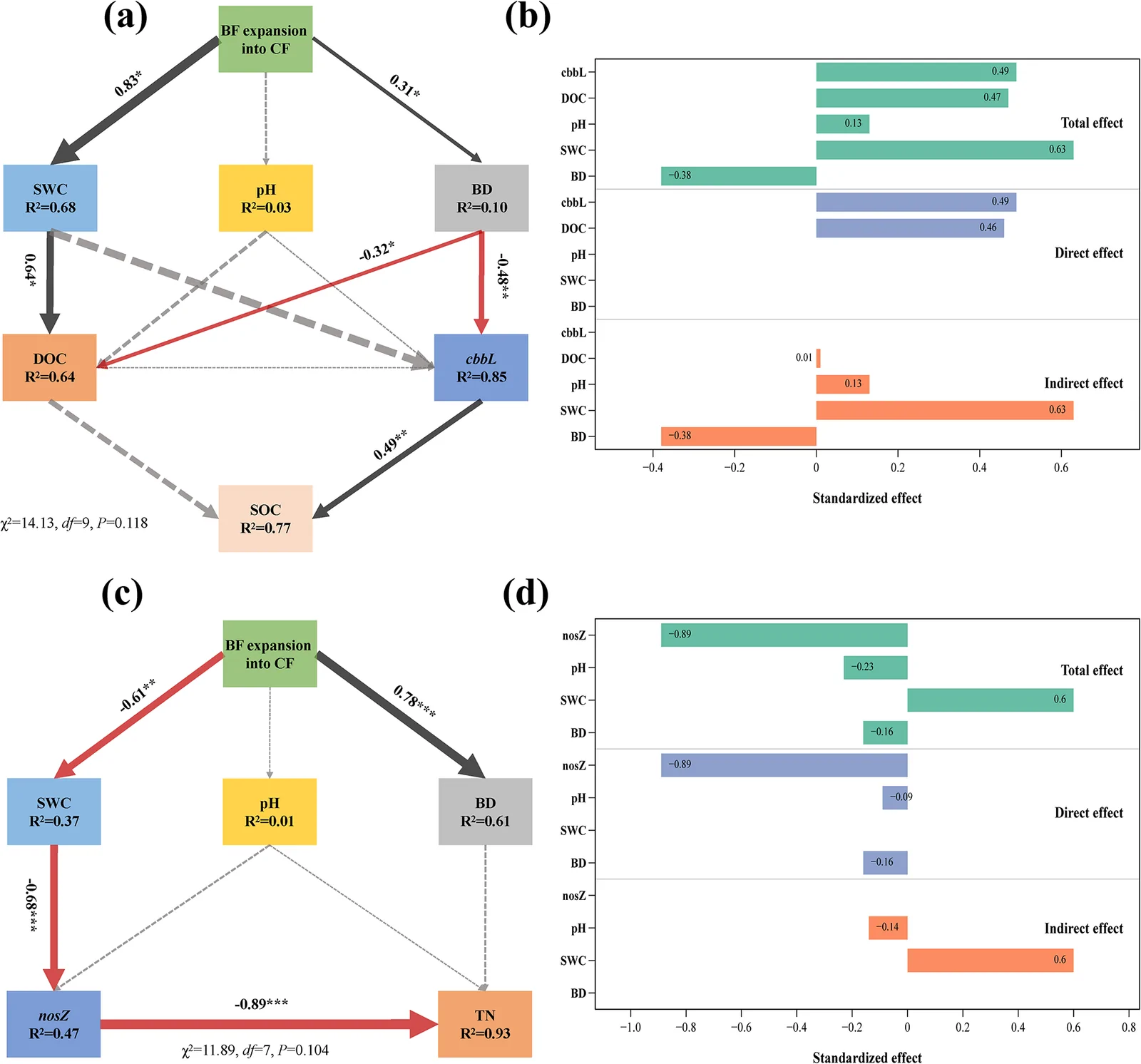
Structural equation modeling (SEM) illustrating the impacts of soil abiotic and biotic properties on SOC **(a)** and TN **(c)** concentrations during BF expansion into CF. Statistical significance was tested using 24 independent soil samples. Solid black and red arrows indicate significant positive and negative effects (*p* < 0.05), respectively, and dashed arrows represent nonsignificant relationships. Values adjacent to arrows are standardized path coefficients, with arrow width proportional to coefficient magnitude. R^2^ donates the proportion of variance explained by the model. Significant levels for predictors: *p < 0.05, **p < 0.01, ***p < 0.001. Standardized total effects of individual drivers on SOC **(b)** and TN **(d)** concentrations derived from the SEM above.

## 4 Discussion

Across the investigated native and bamboo-invaded mixed forest stands, the SOC and TN stocks within the 0–40 cm soil profile ranged from 63.32 to 103.44 Mg ha^-1^ and from 3.08 to 5.52 Mg ha^-1^, respectively (**Table 1**). These baseline values align well with the soil organic pool sizes documented in typical tropical and subtropical forest ecosystems. For instance, Li X. A. et al. (2025) reported that SOC stocks ranged from 56.14 to 70.95 Mg ha^-1^ and TN stocks averaged around 6.35 Mg ha^-1^ within the 0–40 cm profile of a regional forest plantation. Similarly, Du et al. (2014) documented SOC stocks of 78.51 to 92.73 Mg ha^-1^ and TN stocks of 3.42 to 6.31 Mg ha^-1^ (0–40 cm) in subtropical forests on Lushan Mountain. Such consistency confirms that our observed ranges fall well within the typical scope of regional forest ecosystems, validating the reliability of our analytical baseline. Our study highlights that even at the mixed-forest stage of Moso bamboo encroachment (BBLF and BCF), significant depletions in soil organic pools are already evident. The pathways driving these alterations are strictly forest type-specific, pointing to divergent ecological mechanisms between broad-leaved and coniferous ecosystems during the initial phases of bamboo invasion.

### 4.1 Potential pathways underlying soil SOC and TN stock reductions in broad-leaved forests

In agreement with our first hypothesis, substantial depletions of carbon and nitrogen pools occurred across the entire sampled column following Moso bamboo invasion into BLF. These results are consistent with prior regional research (Lin et al., 2018; Xu and Jiao, 2025). However, vertical profile analysis indicated that this decline in soil organic pools was largely restricted to the surface soil layer at a depth of 0–10 cm (**Figures 4a, c**). In broad-leaved ecosystems, the lower initial soil C/N ratio makes the organic matter pool sensitive to external organic inputs (Qin et al., 2017). Moso bamboo encroachment modifies litter chemistry and speeds up forest floor turnover, which is hypothesized to stimulate a strong soil priming effect. This enhanced microbial activity could potentially accelerate the decomposition of older organic matter, ultimately contributing to a net reduction in surface SOC and TN stocks (Qin et al., 2017; Fang et al., 2017). Furthermore, our quantitative decomposition indicated that the reduction in BD accounted for over 60% of the calculated surface stock losses (**Supplementary Table S1**). This suggests that the physical loosening of the topsoil, driven by the expansion of dense bamboo rhizome networks, plays a dominant role in the apparent depletion of organic pools alongside the concurrent decline in element concentrations.

Beyond litter input dynamics, the SEM analysis revealed that variations in SOC concentrations were directly driven by DOC availability, with SWC, soil pH, and BD acting as key indirect regulators (**Figures 5a, b**). Alongside these changes in organic carbon pools, the autotrophic carbon-fixing microbial community was also severely impacted; specifically, the reduction in *cbbL* abundance likely reflects the concurrent degradation of the topsoil microenvironment. The extensive clonal integration of Moso bamboo via rhizome networks produces a dense matrix of fine roots (Kawakami et al., 2022). Such underground reorganization shifts soil physical status, with both BD and SWC dropping markedly as a consequence (**Figures 1a, c**). The resulting drier microenvironment creates osmotic stress and limits the physical diffusion of nutrients (Kour et al., 2024). Concurrently, the significant increase in soil pH (**Figure 1e**), driven by the release of base cations from decomposing bamboo leaves and root secretions (Li et al., 2017; Wu et al., 2024), disrupts the optimal environment for native microbial communities (Lin et al., 2025). Consequently, the depletion of topsoil carbon pools and the suppression of *cbbL-*carrying communities are parallel responses to the degraded physical and chemical microenvironments (**Figure 3a**).

Similarly, the reduction in TN concentrations was significantly associated with shifts in soil BD, whereas nosZ gene abundance showed a nonsignificant path (**Figures 5c, d**). This implies that the decline in TN concentrations is likely driven by a combination of physical soil degradation (e.g., enhanced nutrient leaching due to soil loosening) and reduced litter inputs during initial bamboo expansion, rather than being primarily regulated by the abundance of microbial denitrifiers.

### 4.2 Potential pathways underlying soil SOC and TN stock reductions in coniferous forests

In line with our second hypothesis, Moso bamboo invasion into CF led to substantial reductions in both SOC and TN stocks (**Figures 4b, d**). This outcome contrasts with several previous reports that observed positive or neutral stock changes following bamboo expansion into some coniferous stands (Qin et al., 2017; Wang et al., 2022). Such discrepancies may stem from variations in the specific coniferous forest types, original C/N ratios, or local environmental backgrounds (Wu et al., 2024; Lin et al., 2025). Notably, unlike the topsoil-concentrated depletion observed in broad-leaved forests, the most prominent relative stock losses in coniferous stands were recorded in the sub-soil layer at a depth of 20–40 cm, with SOC and TN stocks exhibiting proportional decreases of 31.2% and 42.8%, respectively (**Figure 4**; **Table 1**). Our quantitative decomposition further highlighted the distinct mechanisms in this deep layer. Specifically, while SOC stock loss was driven jointly by concentration and bulk density changes, the severe sub-soil TN depletion was predominantly driven by concentration decline (accounting for 69.6% of the loss; **Supplementary Table S1**).

Our SEM results demonstrated a significant association between the variance in SOC concentrations and a lower abundance of the *cbbL* gene (**Figures 6a, b**). This concurrent microbial decline reflects the severe subsurface environmental degradation caused by bamboo invasion. Typically, soil microbial proliferation is restricted by the supply of carbon sources and nutrients (Allison and Martiny, 2008; Wan et al., 2021). In these coniferous ecosystems, the extensive root systems of Moso bamboo outcompete and suppress native understory vegetation (Song et al., 2016; Lin et al., 2025). This loss severely disrupts the continuous supply of root exudates and labile organic substrates to deeper soil horizons (Wu et al., 2024). Furthermore, this resource limitation interacts with significant shifts in soil physicochemical properties. On the one hand, the significantly lower BD in the sub-soil (**Figure 1d**) weakens the aggregate-mediated physical protection of organic carbon, exposing legacy native organic matter to accelerated turnover (Six et al., 2002). On the other hand, the significantly drier environment (reduced SWC, **Figure 1b**) restricts the diffusion of DOC, which directly limits substrate availability of native autotrophic microbes (Sinsabaugh et al., 2008). Consequently, the reduction in understory organic inputs and decreased BD are hypothesized to primarily contribute to the macroscopic deep-soil carbon depletion, while these cascading abiotic and biotic stresses collectively suppress the autotrophic carbon-fixing communities, resulting in the parallel decline of *cbbL* gene abundance (**Figure 3b**).

Similarly, the decline in TN concentrations was accompanied by a lower abundance of the *nosZ* gene (**Figures 6c, d**). In the deep soil layer, bamboo roots compete strongly with soil microorganisms for available nitrogen, which directly corroborates our decomposition results showing a massive concentration-driven nitrogen loss (**Supplementary Table S1**). This intense competition restricts substrate availability, which simultaneously depletes the soil nitrogen pool and inhibits the *nosZ*-carrying denitrifying communities (**Figure 3d**). In addition, the significantly drier environment (**Figures 1b, d**) indirectly aggravated the nutrient limitation for deep-soil microbes (**Figure 6**). Therefore, the depletion of deep-soil organic pools in coniferous forests was likely associated with weakened physical protection, strong root competition, and the loss of understory organic inputs. During this process, shifts in soil physical properties intensified the deep-soil degradation, while functional gene abundances acted as sensitive indicators reflecting these environmental changes.

### 4.3 Research limitations and management implications

While this study provides insights into soil carbon and nitrogen dynamics following Moso bamboo expansion, certain limitations inherent to the study design and methodology should be noted. Regarding the experimental design, although the widely used chronosequence approach provides valuable long-term perspectives (Walker et al., 2010; Damgaard, 2019), it inherently uses spatial variation to represent temporal dynamics and assumes adjacent stands shared identical initial soil properties prior to encroachment. Despite our efforts to select plots with consistent site conditions, unmeasured historical land-use differences could still exist. Furthermore, our sampling and measurement approaches present certain constraints. Soil sampling was conducted only in July, capturing a summer snapshot that may not reflect seasonal variations (Sardar et al., 2023). Additionally, soil stocks were calculated using a fixed-depth approach without gravel correction because coarse fragment volume was not recorded, which may introduce some estimation bias. Finally, measuring *cbbL* and *nosZ* abundance via DNA templates indicates the genetic potential rather than active *in-situ* transcriptional activity of the soil microbiota (Carini et al., 2016). Future studies should combine long-term field monitoring, isotope tracing techniques (to explicitly test the hypothesized priming effects), and metatranscriptomics to better understand real-time physical and microbial functions.

Despite these limitations, our findings provide practical implications for targeted forest management. For broad-leaved ecosystems, bamboo expansion mainly leads to surface soil drying and structural loosening (**Figures 1a, c**). Therefore, implementing early and low-intensity thinning of Moso bamboo is recommended to protect the topsoil structure and maintain moisture retention (Li et al., 2018; Zhang et al., 2024). In contrast, in coniferous stands, the loss of SOC and TN stocks is primarily driven by weakened physical protection and the loss of understory carbon inputs, which were caused by aggressive bamboo rhizome expansion. Accordingly, forest management in these coniferous plantations should focus on preserving understory vegetation biodiversity (Nilsson and Wardle, 2005; Gilliam, 2007). Maintaining a diverse understory layer can provide continuous organic matter to the sub-soil and reduce belowground physical disturbances, which will help alleviate the depletion of deep-soil carbon and nitrogen pools.

## 5 Conclusions

In conclusion, our observations during the summer growing season indicate that Moso bamboo expansion is consistently associated with the depletion of SOC and TN stocks in adjacent BLF and CF through distinct depth-dependent patterns. In the BLF, carbon and nitrogen losses are concentrated in the topsoil, which are hypothesized to be linked to altered litter chemistry and the physical degradation of the surface microenvironment. In the CF, however, the depletion is most prominent in the sub-soil layer. This deep-soil degradation is potentially associated with weakened physical protection, strong root competition, and the loss of continuous understory carbon inputs. Concurrently, the decline in functional microbial genes (*cbbL* and *nosZ*) serves as a sensitive potential biological indicator of these environmental stresses. While multi-season studies are needed to capture temporal dynamics, these findings suggest that managers should implement forest-type-specific strategies to mitigate soil degradation. Furthermore, incorporating belowground functional genes as ecological indicators could improve future plant invasion risk assessments in subtropical forest ecosystems.

## Data Availability

The raw data supporting the conclusions of this article will be made available by the authors, without undue reservation.
