# Supplementary material for: Vertically distinct patterns of soil carbon and nitrogen stock depletion associated with Moso bamboo encroachment into subtropical forests
Source: Front Plant Sci. 2026 Sep 30;17:1954514. doi: 10.3389/fpls.2026.1954514 (PMC13661586; doi:10.3389/fpls.2026.1954514)
Supplement: Supplementary file 1 [file Table1.docx]

**Table S1.** Decomposition of SOC and TN stock losses into concentration and bulk density components for each soil depth and forest conversion type.

| **Forest conversion** | **Soil depth (cm)** | **SOC stock loss (Mg·ha^-1^)** | **SOC loss contribution (%)** | | **TN**  **stock loss (Mg·ha^-1^)** | **TN loss**  **contribution (%)** | |
| --- | --- | --- | --- | --- | --- | --- | --- |
|  |  |  | **Concentration** | **BD** |  | **Concentration** | **BD** |
| **BLF → BBLF** | 0–10 | 19.32 | 37.1 | 62.9 | 0.98 | 32.6 | 67.4 |
|  | 10–20 | 7.93 | 25.8 | 74.2 | 0.28 | −9.2 | 109.2 |
|  | 20–40 | 6.96 | 60.5 | 39.5 | 0.42 | 64.3 | 35.7 |
| **CF → BCF** | 0–10 | 4.74 | 37.4 | 62.6 | 0.51 | 44.7 | 55.3 |
|  | 10–20 | 5.64 | 51.3 | 48.7 | 0.44 | 53.4 | 46.6 |
|  | 20–40 | 11.9 | 49.5 | 50.5 | 0.98 | 69.6 | 30.4 |

Note: Abbreviations: SOC, soil organic carbon; TN, total nitrogen; BD, bulk density; BLF, evergreen broad-leaved forest; BBLF, bamboo-broad-leaved mixed forest; CF, coniferous forest; BCF, bamboo-coniferous mixed forest.
